# Biosensors for Non-Invasive Detection of Celiac Disease Biomarkers in Body Fluids

**DOI:** 10.3390/bios8020055

**Published:** 2018-06-16

**Authors:** Tibor Pasinszki, Melinda Krebsz

**Affiliations:** 1 Department of Chemistry, School of Sciences, College of Engineering, Science & Technology (CEST), Fiji National University, P.O. Box 7222 Nasinu, Fiji; 2 Institute of Chemistry, Faculty of Science, ELTE Eötvös Loránd University, Pázmány P. sétány 1/A, H-1117 Budapest, Hungary; melinda.krebsz@gmail.com

**Keywords:** celiac disease, gluten, biomarkers, biosensors, anti-gliadin, anti-deamidated gliadin, anti-transglutaminase, HLA alleles

## Abstract

Celiac disease is a chronic gluten-initiated autoimmune disorder that predominantly damages the mucosa of the small intestine in genetically-susceptible individuals. It affects a large and increasing number of the world’s population. The diagnosis of this disease and monitoring the response of patients to the therapy, which is currently a life-long gluten-free diet, require the application of reliable, rapid, sensitive, selective, simple, and cost-effective analytical tools. Celiac disease biomarker detection in full blood, serum, or plasma offers a non-invasive way to do this and is well-suited to being the first step of diagnosis. Biosensors provide a novel and alternative way to perform conventional techniques in biomarker sensing, in which electrode material and architecture play important roles in achieving sensitive, selective, and stable detection. There are many opportunities to build and modify biosensor platforms using various materials and detection methods, and the aim of the present review is to summarize developments in this field.

## 1. Introduction

Celiac disease (CD) is a genetically predisposed chronic immune-mediated enteropathy that effects about 1% of the population of Europe and North America [1,2,3]. CD is caused by the ingestion of some peptides derived from wheat, barley, rye, oats, and hybrids of these grains, and intestinal and extraintestinal symptoms usually last for days, weeks, months, or even years after ingesting gluten. Although CD is possibly as old as human history [4,5], it has become the disease of our modern age due to increased consumption of gluten in food. CD has numerous symptoms; however, none of them is specific, thus a large percent of CD patients are misdiagnosed with other disorders. Tests for the diagnosis of CD are currently based on biopsy, genetic analysis of human leukocyte antigen (HLA) DQ genes, and serological markers. CD predominantly affects and damages the mucosa of the upper small intestine, therefore repeated intestinal biopsy (typically three to five times) and histopathologic judgment of the tissue are required for the final diagnosis of CD [5,6]. Biopsy, however, is invasive and cannot be routinely and frequently applied. A large part of the genetic risk of developing CD is due to the presence of HLA class II alleles [5,7]. HLA-DQ2 and HLA-DQ8 have been found to exhibit the strongest association with CD. Although the absence of these genes is a reliable negative predictor of CD, their presence is not sufficient for the positive diagnosis of CD. CD-specific antibodies are produced in the intestinal mucosa upon gluten exposure and bind to their specific antigen in the diseased mucosa and appear in the blood [5,8]. The detection of these antibodies in blood provides an essential route for non-invasive identification of CD; however, their presence in blood depends on gluten intake. A successful gluten-free diet results in slow elimination of CD-specific antibodies from blood, therefore, antibodies can act as biomarkers of the untreated disease, and can be used for follow-up of clinical treatment and adherence to the gluten-free diet. All three biopsy, genetic analysis, and serological markers have their limitations concerning applicability, effectiveness, and cost, therefore their combined application is required. Serological markers, however, provide the possibility for non-invasive screening of symptomatic patients before biopsy and for population screening. Several clinical tests were developed in the past to determine serological biomarkers based on immunofluorescence (IF) and enzyme-linked immunosorbent assay (ELISA) [9,10,11]. Limitation of these traditional assay methods for their wide scale routine application is that they require qualified operators and laboratory facilities equipped with expensive and sophisticated instruments, and they are time-intensive thus results are available only after a time delay. The development of sensitive, rapid, and simple immunoassay methods for CD-biomarker detection in blood therefore has a great diagnostic value. Electrochemical and optical biosensors are highly attractive for detecting biomarkers due to their high sensitivity and selectivity, relatively easy fabrication and operating procedures thus low cost, the potential to be miniaturized, and simplicity for operators [12,13]. They appear as promising alternative to conventional ELISA techniques. In addition, these biosensors have also the potential to provide basic tools for point-of-care (POC) testing (testing at or near the site of patient care). The first CD biosensor was developed in 2007 [14], and since there is an enormous interest for developing CD sensors for clinical diagnosis and POC testing. The aim of the current review is to summarize recent developments on this field focusing on sensor architectures.

## 2. Biomarkers of CD

CD is a unique disease in a sense that its trigger (gluten) is identified, and it has serological markers [5,15]. Upon gluten exposure, disease-specific antibodies are produced in the intestinal mucosa of CD patients and appear in the diseased intestinal mucosa, saliva, and blood. The latter is the basis of their serological detection. These CD-specific antibodies, namely anti-gliadin antibody (AGA), antibody against deamidated gliadin peptides (DGPA), endomysial antibody (EMA), and transglutaminase-antibody (TGA), are directed both against components of gluten (gliadin) and against components of the tissue, e.g., tissue transglutaminase (tTG). They belong to the immunoglobulin (Ig) classes A and G. Both IgA and IgG tests are used, but IgG tests must be applied for CD patients with selective IgA deficiency. Although AGA tests have their historical value in CD detection, they are no longer routinely recommended [16,17] because of their lower sensitivity and specificity than EMA, DGPA, and TGA tests. The overexpression of the regenerating gene Iα (REG Iα) in small bowel of CD patients has an important role in the regeneration and survival of target cells, and REG Iα emerged recently as a new biomarker of CD [18]. T cells has a central role in CD in causing tissue destruction [19] and serum T cells can be used for CD diagnostic purposes. Biosensors for the detection of REG Iα and CD-specific T cells in blood have not been developed to date. Although only a fraction of the population which has inherited the HLA-DQ2 and/or HLA-DQ8 genes suffers from CD, the identification of these genes in blood is important for ruling out CD or suggesting further clinical tests. It must be noted that selectivity and sensitivity of biomarker detection are varied, and CD is a complex disorder. Positive serological results strongly support the diagnosis of CD, but negative results do not rule out the possibility of CD. Biomarker test are positive only if CD-patients are on gluten-containing diet. It was observed that the prevalence of seronegative CD accounts for up to 10% of all diagnosed CD cases [20]. Therefore, biomarker detection must be followed by genetical testing and biopsy before a conclusion on CD is drawn.

## 3. Biosensors for CD Detection

Biosensors in general are composed of a bioreceptor and a transducer in tight connection, and a signal-processing unit [12,13,21]. The bioreceptor recognizes the analyte by binding to the analyte, and the biochemical signal generated upon this event is converted to an electronic, optical, gravimetric, magnetic, etc., signal by the transducer and conveyed toward the signal-processing unit where it is amplified and processed. A secondary receptor with a label is often required to amplify the microscopic changes accompanying the receptor-analyte complex formation. The selectivity of the sensor is therefore determined by the bioreceptor. In the case of CD biomarkers, it is straightforward to apply the antigen of the biomarker as the bioreceptor because antigen-antibody interactions are specific. Sensitivity of the sensor is strongly determined by the transducer. Biosensor platforms constructed for CD-biomarker sensing to date include electrochemical, mass-sensitive, or optical transducing elements which can generate measurable current, frequency, or light signals for signal-processing units. Electrochemical biosensors are most widely constructed due to their simplicity and sensitivity. Considering the latter, increasing the electron transfer toward the electrode is of crucial importance. For increasing the electrode response magnitude, incorporating conducting materials into the electrode modifying thin film layer, increasing the electrode surface, and/or catalyzing the redox reaction are generally applied. Wide range of materials can be used for this purpose, including inorganic and organic substances, polymers, and nanomaterials, what provides large variability for sensor architectures. All of these, the construction of the transducer and anchoring bioreceptor onto the transducer surface, make biosensor fabrication an interesting chemical challenge.

### 3.1. Detection of Anti-Gliadin Antibody (AGA)

A sandwich-type amperometric immunosensor that mimics traditional ELISA type architecture was constructed by Rosales-Rivera et al. for the detection of AGAs in human sera [22]. The analyte recognition of the sensor was based on gliadin antigen–AGA interaction and signal amplification was achieved by using anti-human IgA and anti-human IgG labeled with horse radish peroxidase (HRP) antibodies. The working electrode of the sensor was fabricated by controlled anchoring of gliadin antigens on gold electrode using a carboxylic-ended bipodal alkanethiol (22-(3,5-bis((6-mercaptohexyl)oxy)phenyl)-3,6,9,12,15,18,21-heptaoxadocosanoic acid (DT2)). DT2 was chemisorbed on the gold surface and covalently linked with amino groups of the antigen protein using the standard *N*-(3-dimethylaminopropyl)-*N*-ethyl-carbodiimide (EDC)/*N*-hydroxysuccinimide (NHS) method for carboxyl group activation. The immunosensor exhibited stable and reproducible low limits of detection (LOD) and allowed the estimation of semi-quantitative AGA content (Table 1). An excellent correlation was obtained between results provided by the immunosensor and ELISA. Ortiz et al. [23] developed a sandwich-type biosensor for the detection of IgA and IgG AGA in real samples of CD patients under follow-up treatment. The sensor was based on thiolated β-cyclodextrin polymer (CDPSH) modified gold electrodes and on the supramolecular self-assembly of adamantane-carboxymethylcellulose-gliadin (ADA-CMC-GLI) polymers on cyclodextrin surfaces. Mouse anti-IgG-HRP conjugate was used as target receptor and label. The constructed sensor exhibited high reproducibility, an order of magnitude lower detection limit (20 ng/mL), and shorter assay time (~40 min vs. >3 h) than the reference ELISA. A similar AGA sensor based on host/guest interactions between adamantane moieties of the modified sensor surface and β-cyclodextrin hosts using ADA-CMC-GLI was constructed by Wajs et al. [24]. The working electrode of the amperometric immunosensor was fabricated consecutively by electrodepositing a polypyrrole (PPy) layer on gold surfaces of disposable screen-printed electrode (SPE) using 1*H*-pyrrole-1-propanoic acid, activating carboxyl groups by EDC/NHS, covalently binding mono-6-amino-6-deoxy-β-cyclodextrin (CDAM), and attaching ADA-CMC-GLI via host/gest interaction. Amperometric signal was amplified using anti-IgG-HRP conjugate. Three other sensors with three alternative ways of immobilizing the gliadin antigen were also prepared. Gliadin was directly attached to the Au/PPy surface (Au/PPy/GLI) using EDC/NHS in the first case, gold electrodes were modified with a self-assembled monolayer (SAM) of hepta-6-thio-6-deoxy-βCD11 onto which the ADA-CMC-GLI polymer was self-assembled (Au/CD/ADA-CMC-GLI) in the second case, and gliadin was immobilized on a SAM of 3-mercaptopropionic acid thus lacking both PPy and CD moieties (Au/C3SAM/GLI) in the third case. Sensitivities and LOD values of all these three sensors were significantly lower than that of Au/PPy/CD/ADA-CMC-GLI (0.45 µA∙mL/µg and 33 ng/mL, respectively) notwithstanding that the gliadin surface coverage was similar, indicating the importance of PPy-CD-ADA interaction. The fabricated sensor exhibited very good signal recovery, near 100%, in spiked serum samples.

Fiber-optic biosensors have an advantage of high detection speed thus they are widely investigated for sensor applications. Corres et al. [25] constructed an AGA immunosensor based on long-period fiber grating (LPFG) transducer. The fiber was modified by a precursor layer of silica-nanospheres using the electrostatic self-assembly technique based on poly(allylamine hydrochloride) (PAH) and LUDOX^®^ SM-30 SiO_2_ water colloidal suspension as polycation and anionic species, respectively, and gliadin was deposited onto the highly porous precursor layer as target recognition element. Corres et al. [26] developed a similar optical fiber (OF) AGA immunosensor where gliadin antigens were immobilized onto surfaces of tapered optical fibers using the electrostatic self-assembly technique and gliadin as the cationic and poly(sodium 4-styrenesulfonate) (PSS) as the anionic species. The sensor exhibited a LOD of 1 ppm, fast response (100 min), and real time detection. Layer-by-layer (LbL) assembly of oppositely charged material sheets is widely used to fabricate functional thin films with well-defined thicknesses for sensor applications [27]. Socorro et al. [28] constructed an AGA sensor based on thin-film-coated tapered single-mode optical fibers (T-SMFs). The sensing layer of the sensor was prepared by depositing PAH, PSS, and poly(acrylic acid) (PAA) on T-SMFs by LbL assembly to obtain a multilayer film with a sequence of (PAH/PAA)(PAH/PSS)_3_(gliadin/PSS)_2_(gliadin). The sensor was able to detect AGAs concentrations of 5 ppm.

### 3.2. Detection of Antibody against Deamidated Gliadin Peptides (DGPA)

The first electrochemical immunosensor for the detection of DGPA was developed by Neves et al. in 2013 [29] (Table 1). The sensor is based on a fusion protein of four deamidated gliadin peptide (DGPx4: carrier-33merDGP-26merDGP-DQ2-g1-DQ2-g2) as capture element. To construct the sensor, a disposable screen-printed carbon electrode (SPCE) was modified with carboxylated multi-walled carbon nanotubes (MWCNTs) by drop coating followed by in situ electrochemical deposition of gold nanoparticles (AuNPs). DGPx4 capture peptides were deposited on the modified surface from solution and free surface sites were blocked with bovine serum albumin (BSA). The detection of DGPA was based on sandwich-type strategy using anti-human IgG labeled with alkaline phosphatase (AP) as signal labels and on enzymatic deposition of metallic silver catalyzed by AP. Real serum samples were successfully assayed and results were corroborated with reference ELISA.

### 3.3. Detection of Anti-Transglutaminase Antibody (TGA)

**Figure 1:**
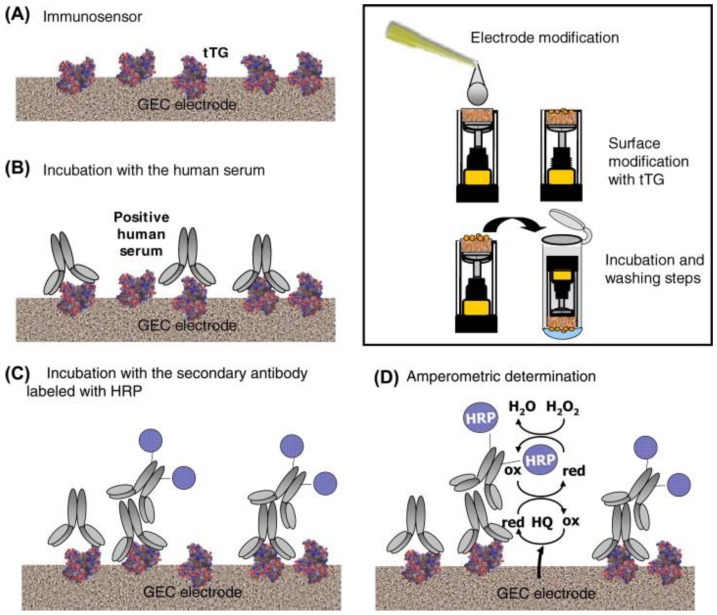
Schematic representation of steps of the construction of a TGA immunosensor [30]: (**A**) adsorption of tTG on the bare GEC electrode; (**B**) immunological reaction of tTG with serum TGA; (**C**) immunological reaction of TGA with secondary antibody; and (**D**) amperometric determination. Inset shows electrode modification and washing steps. Copyright 2009. Reproduced with permission from Elsevier.

The first electrochemical TGA immunosensor for detecting both IgG and IgA type antibodies in human sera was developed by Balkenhohl and Lisdat [14] in 2007. The sandwich-type impedimetric sensor was constructed by immobilizing transglutaminase (TG) on screen-printed gold electrodes (SPGE) using PSS binding layers and blocking unspecific and residual binding sites with bovine serum albumin (BSA). The signal was amplified using HRP-labeled secondary immunoglobulins and applying subsequent biocatalytic oxidation of 3-amino-9-ethylcarbazole (AEC). The constructed sensor was able to detect TGA in human sera, and assist in the diagnosis of celiac disease, but measured TGA concentrations did not correspond completely to reference ELISA measurements. Pividori et al. [30] constructed a sandwich-type amperometric TGA immunosensor by immobilizing tissue TG (tTG) from guinea pig liver on graphite–epoxy composite (GEC) electrode and applying secondary enzyme-labeled antibodies (HRP-conjugated goat IgG fraction to human IgG, IgA, and IgM, human IgA (alpha chain), and human IgG Fc) for signal amplification. Steps of the construction of the sensor are illustrated in Figure 1. The HRP-conjugated goat IgG fraction to human IgA (alpha chain) was proved to be the best secondary antibody in terms of reproducibility for the prediction of a positive serum. The constructed sensor exhibited a 70, 100, and 100% sensitivity, specificity, and positive predictive value, respectively, compared with ELISA. Dulay et al. [31] developed an amperometric TGA immunosensor consisting of gold-based self-assembled monolayer (SAM) of a carboxylic group terminated bipodal alkanethiol, DT2, that was covalently linked to tissue transglutaminase using EDC/NHS. The presence of the TGA autoantibodies was recorded using HRP-labeled anti-human-IgG reporter antibody receptors. The immunosensor exhibited stable quantitative response to antibodies, and comparison of results with those of reference ELISA showed an excellent degree of correlation. A disposable voltammetric immunosensor for the detection of IgA and IgG type TGA in real patient’s samples was constructed by Neves et al. [32] by modifying screen-printed carbon electrodes (SPCE) with multi-walled carbon nanotubes (MWCNT) and gold nanoparticles (AuNP), and immobilizing tTG on the nanostructured electrode surface using drop-coating. A sandwich-type strategy was applied for autoantibody detection using alkaline phosphatase (AP) labeled anti-human IgA or IgG antibodies, and anodic redissolution of enzymatically generated silver was used for generating the analytical signal. The sensor exhibited good reproducibility (evaluated for two interday immunosensing assays using three electrodes), and results were corroborated with ELISA.

Kergaravat et al. [33] developed an electrochemical magneto immunosensor for the detection of TGA Type 2. TG Type 2 was immobilized covalently on magnetic beads (MB) modified with tosyl groups and the immunological reaction was performed on these MB-TG2 beads as solid support. Anti-human IgA(α-chain specific)-HRP was used as receptor and signal label in a sandwich-type arrangement. Beads were collected magnetically onto the surface of a screen-printed electrode (SPE) for square wave voltammetric measurements. The sensor exhibited 84% specificity and relatively rapid biomarker detection compared with the reference serological method based on indirect immunofluorescence for anti-endomysial antibodies. A miniaturized biosensor for TGA IgG detection in human sera was constructed by Martín-Yerga et al. [34] based on in situ detection of CdSe/ZnS quantum dots (QDs). The sensor was fabricated by adsorbing tTG on an 8-channel SPCE, followed by a blocking step using β-casein. Anti-human IgG QDs were applied as receptors and signal labels. The developed immunosensor exhibited good reproducibility (based on two interday assays and three electrodes), high stability, and proved to be a trustful screening tool for CD diagnosis discriminating between positive and negative sera samples. Sensors were stable for one month at 4 °C.

An impedimetric TGA immunosensor based on interdigitated electrode (IDE) arrays with nanometer scale gaps and AuNPs as signal enhancement labels was constructed by Singh et al. [35]. IDE was fabricated using a combination of UV photolithography and electron-beam lithography, and SAMs of cysteamine were deposited on the gold surface. tTG antigens were covalently linked to cysteamine using glutaraldehyde, and nonspecific binding sites were blocked by polyvinylpyrrolidone (PVP). For nanoparticle-based signal enhancement, AuNPs conjugated with protein-A, a staphylococcal membrane protein that binds very specifically with the Fc region of IgG, (Pr-A-AuNP) could bind with captured antibodies. Labeling improved the sensitivity of the sensor by about 350%; however, the LOD did not change much. Labeling increased the sensitivity of the sensor to the level of on-chip ELISA. A sensitive label-free impedimetric TGA immunosensor was developed by West et al. [36] based on overoxidized polypyrrole (OPPy) as substrate for binding capture tTG protein. The working electrode of the sensor was constructed by electrodepositing OPPy and AuNPs on glassy carbon electrode (GCE) and drop-coating tTG onto the AuNP/OPPy film. The sensor was stable for one day, but sensor stability gradually decreased about 9% compared to its initial values after 14 days of storage. A similar sensor was constructed by Wilson et al. [37] but using Pt as the working electrode. The Pt electrode was modified by electrodeposition of a PPy–cobalt (II) salicyladiimine metallodendrimer (Co(II)SDD) composite film, and tTG and BSA were deposited on the composite surface consecutively from solution as target capture element and blocking agent of nonspecific binding, respectively. The construction of the sensor was simple, but LOD was two orders of magnitude higher (201 vs. 5.2 ng/mL) than that of GCE/OPPy/AuNP-based immunosensor. A blocking-free immunosensing and a one- and a multi-step labeling strategy for TGA detection was developed by Martín-Yerga and Costa-García [38]. The sensor is constructed by depositing human tTG on 8-channel SPCE by drop-coating. The multi-step labeling was performed by first depositing biotinylated anti-human IgA (anti-IgA-BT) and then a CdSe/ZnS quantum-dot streptavidin conjugate (QD-STV) on target modified sensor surfaces. In the case of one-step methodology, a mixture of target TGA, anti-IgA-BT, and QD-STV was deposited simultaneously from solution. The one-step strategy was much simpler and faster, but the multi-step strategy provided superior sensor performances including higher sensitivity, lower LOD and wider linear detection range. The sensor fabricated with one-step strategy, however, was able to differentiate the clinical relevant concentrations and performing 8 simultaneous analyses in less than 90 min. The sensors exhibited good reproducibility and stability over a one-month testing period. A similar TGA sensor using the multi-step strategy and identical sensor construction steps, but magnetoelectrochemical support for SPCE was developed by Martín-Yerga et al. [39]. The magnetoelectrochemical support improved significantly the analytical performance of the sensor, e.g., LOD was 1.0 and 1.7 U/mL at the presence and absence of magnetic field, respectively.

Carbon nanomaterials emerged recently as promising elements of sensor platforms [27], and the first graphene quantum-dot (GQD)-based TGA immunosensor was prepared in 2017 by Gupta et al. [40]. The sensor was fabricated by modifying the MWCNT-AuNP working electrode of a MWCNT-AuNPs modified SPCE by chemisorbing cysteine onto AuNPs and covalently linking GQDs to cysteine via EDC/NHS coupling. Polyamidoamine (PAMAM) dendrimers were attached to GQD through carbodiimide coupling and tTG antigens were anchored to PANAM using EDC/NHS. The label free sensor exhibited ultrahigh sensitivity (1297.14 µA/cm^2^/pg), high selectivity, and low detection limit of 0.1 fg IgA isotype TGA in 6 μL sample. It was able to detect TGA from one drop (6 μL) of blood in 20 min.

Surface immobilization of target capture antigens on electrode surfaces usually involves several synthetic steps, thus they are time-consuming and costly. Rosales-Rivera et al. [41] developed a method for direct immobilization of tTG on gold surfaces of electrodes by chemically modifying the antigenic protein. Introducing disulfide groups through amine moieties of tTG preserved the tTGs antigenic property, but it was lost when disulfide groups were introduced through carboxylic or hydroxyl groups. The immunosensor for TGA detection was prepared by co-self-assembly of modified tTG and poly(ethylene glycol) alkanethiol on gold disk electrodes and used for the detection of IgA and IgG autoantibodies. HRP-labeled anti-idiotypic antibodies were used as receptors and signal labels. The constructed sensor was simple, rapid, and exhibited high stability over a period of two months. Assay time was short, 30 min.

A sandwich-type electrochemiluminescence (ECL) TGA immunosensor with wide dynamic range of 1–10,000 ng/mL TGA and low LOD of 0.5 ng/mL was developed by Habtamu et al. [42] for CD diagnosis in human serum. The sensor was based on membrane-templated gold nanoelectrode ensembles, tTG target capture elements immobilized on polycarbonate (PC) surface of the templating membrane, and a streptavidin (SA)-modified ruthenium-based ECL label attached to biotinylated secondary IgG antibodies. The novel sensing strategy was thus based on spatial separation of the electrochemical reaction at the Au electrode from the biorecognition event (ECL-emitting region). The sensor was shown to be capable of discriminating between healthy and CD patients.

The surface plasmon resonance (SPR) is a very sensitive technique for investigating metallic layer/dielectric medium interfaces. A low cost SPR immunobiosensor was constructed by Cennamo et al. [43] for the diagnosis and/or follow-up of CD. The sensor was fabricated by removing the cladding of a plastic optical fiber (POF) along half the circumference, sputtering a thin gold film onto the bare fiber, and depositing guinea pig liver tTG as target recognition element. The sensor was able to detect TGA in the nanomolar range, far below that occurs in CD patients’ blood.

The first piezoelectric immunosensor for detecting CD biomarkers was developed in 2014 by Manfredi et al. [44]. The TGA immunosensor was constructed by modifying quartz crystals by constructing SAMs of 11-mercaptoundecanoic acid (MUA) on gold surfaces and covalently linking open-tTG to activated carboxyl group of MUA using EDC/NHS coupling. TGA was detected in a sandwich-type arrangement using anti-IgG-AuNP receptors. The sensor developed was suitable for TGA determination in serum matrix.

### 3.4. Detection of HLA-DQ2/DQ8 Alleles

**Figure 2:**
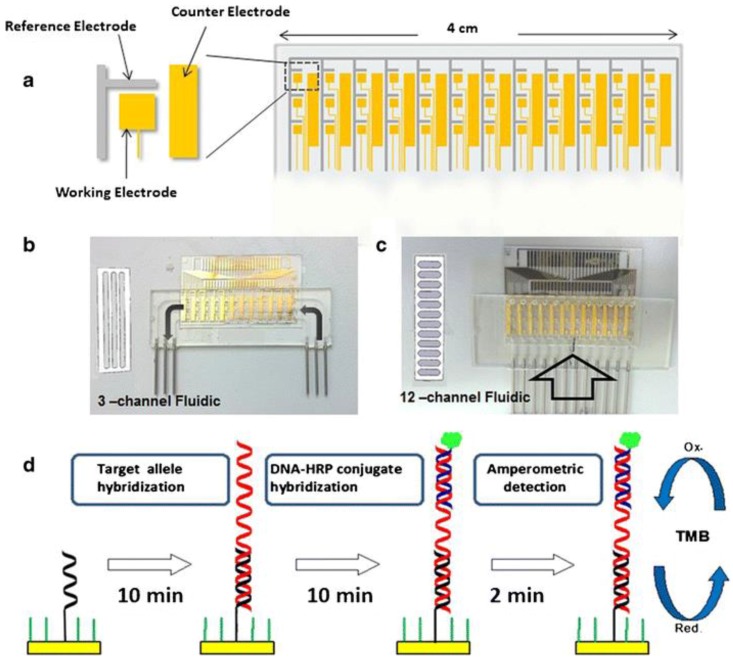
Schematic of the electrochemical genosensor array: (**a**) the 36-electrode array and a zoomed view of the electrode arrangement; (**b**,**c**) electrode array after mounting within 3- and 12-channel fluidic cells; (**d**) steps involved in genosensor assay [45]. Copyright 2014. Reproduced with permission from Springer-Verlag.

CD patients carry either an HLA-DQ2 encoded by the DQA1*05:01/05:05, DQB1*02:01/02:02 alleles or HLA-DQ8 heterodimer encoded by the DQA1*03:01, DQB1*03:02 alleles, therefore detection of these alleles in blood is of crucial importance in determining CD predisposition. Joda et al. [45] developed a multiplex electrochemical genosensor for rapid medium-high resolution HLA-DQ2/DQ8 genotyping. The sensor construction involved (1) the fabrication of an electrode array chip on a glass substrate using standard photolithographic methodology; (2) fabrication of a set of dedicated fluidic cells by high-precision milling of a polycarbonate substrate; (3) creation of fluidic channels by precise laser cutting of double-sided medical-grade adhesive foil; (4) sandwiching this layer between the electrode array and the fluidic cell in order to create fluidic channels; and (5) anchoring target recognition elements on electrode surfaces (see Figure 2).

The microfluidic channels of the sensor were protected from nonspecific adsorption of HRP-labeled oligonucleotides by cross-linking to a PEG-based monomer. HLA alleles were detected using a Polymerase Chain Reaction (PCR) approach. Target complementer thiolated ssDNA probes were co-immobilized on gold electrode surfaces with alkanethiol spacers first, and after target allele hybridization, target complementer HRP-modified single stranded DNA (ssDNA) reporter probes were immobilized on modified electrode surfaces by hybridization. The DNA probe functionalized electrode array was estimated to have high real time (4 °C) storage stability for at least 2 years and exhibited good discrimination capacity of the proposed probes. An HLA genosensor with similar design but using sequence specific primer that produces a PCR product flanked by two ssDNA tails was constructed by Joda et al. [46]. These two ssDNA tails were used for hybridization with thiolated ssDNA probes immobilized onto the surface of electrode array and with HRP-labelled reporter ssDNA probe. The sensor exhibited fast response and targets could be detected in 5 min.

Beni et al. [47] developed a turn-on fluorescence genosensor for the identification of HLA-DQ2 genes using molecular-beacon (MB)-functionalized AuNPs. MBs, consisting of a 17-nucleotide-long recognition sequence, a terminating thiol group, and a five-nucleotide stem with ten thymines between the functional elements of the MB, were especially designed for target recognition. They were modified at their 5′ end with the fluorophore Rhodamine 6G or ATTO 647N and at their 3′ end with a thiol group and anchored to AuNPs using chemisorption. MBs are in closed configuration in the absence of target DNA, thus their fluorescence is quenched by AuNPs. Upon hybridization with the target sequences, however, fluorophores move away from AuNPs thus fluorescence turns on. AuNPs functionalized with two molecular beacons allowed the low-resolution typing of the DQ2 gene at the nanomolar level.

## 4. Conclusions and Outlook

**Figure 3:**
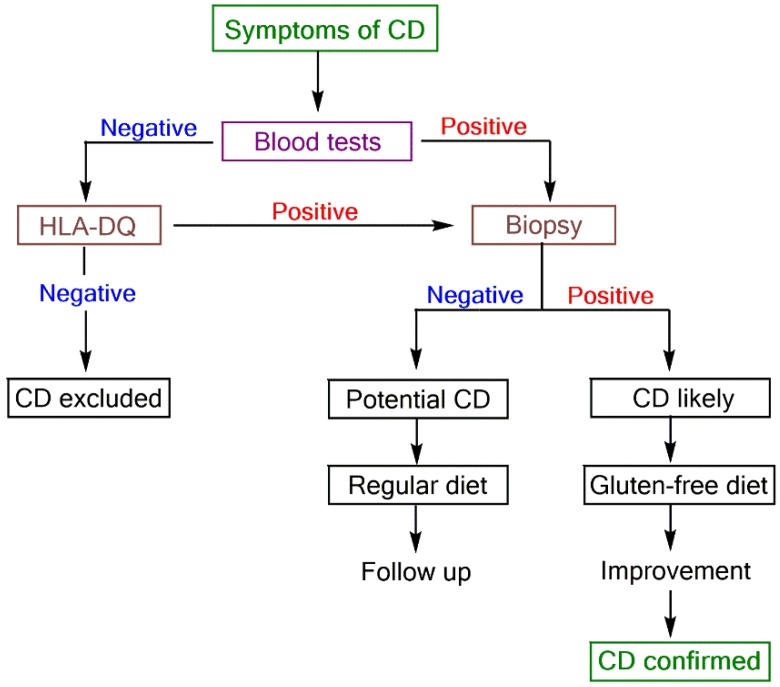
Proposed scheme for CD diagnosis [5].

CD is difficult to diagnose because its diagnosis is complex and tends to require a high level of clinical suspicion [5]. CD is a complex disorder with various extraintestinal manifestations, and a clinical test that can unambiguously diagnose or exclude celiac disease in every individual is not developed to date. Up to now, only the combined analysis of biopsy, symptoms, and laboratory test results can confirm the diagnosis of CD. A proposed diagnostic scheme to diagnose CD is shown in Figure 3 [5].

Blood tests are important starting points of CD diagnosis because they are non-invasive and provide information on possible CD existence in relatively short time. Therefore, individuals possessing unexplained signs or symptoms of CD or genetically predisposed should undergo CD serological screening. There is a high demand for developing simple, fast, efficient, reliable, robust, portable, user-friendly, and cost-effective analytical tools for CD diagnosis and point-of-care testing. Biosensors are challenging conventional ELISA techniques in CD diagnosis and monitoring because they have the potential to fulfill all these requirements. Although the history of CD biosensors goes back only a decade, numerous immunosensors incorporating various sensor platforms have been developed for CD biomarker detection. Although it is too early to recommend which detection method is the most appropriate, because many parameters must be considered including the analyte, sample matrix, assay format, labelling requirements, miniaturization, and cost, simplifying the sensor architecture points toward faster and cheaper production and application. Sensor platforms can be varied in many ways and there is quest for increasing sensitivity, selectivity, reproducibility, and stability, as well as developing platforms for simultaneous detection of more than one biomarker for increasing predictive value of blood tests. Although biosensors are expected to move into the field of routine clinical application, currently, they are at the advanced laboratory stage. CD biosensors developed so far are not yet validated using large numbers of patient samples, and interestingly, specificity studies are rarely conducted. Studies for possible mass production, including the production of identical sensor batches and scaling-up, have not been carried out. There is now clearly an intention in medical practice to avoid unnecessary invasive intestinal biopsy, and biosensors will have an important and vital role in CD diagnosis and monitoring in the future.

## Figures and Tables

**Table 1:** CD biosensors.

Sensor Platform//Label	Analyte	Instrumental Technique ^1^	Linearity Range	LOD	Ref.
Au/DT2/anti-gliadin//anti-IgG-HRP	AGA	AMP	0–1000 ng/mL	46 ng/mL	[22]
Au/CDPSH/ADA-CMC-GLI//anti-IgG-HRP	AGA	AMP	0–750 ng/mL	20 ng/mL	[23]
Au/PPy/CD/ADA-CMC-GLI//anti-IgG-HRP	AGA	AMP	0–10 µg/mL	33 ng/mL	[24]
Au/PPy/GLI//anti-IgG-HRP	AGA	AMP	0–10 µg/mL	135 ng/mL	[24]
Au/CD/ADA-CMC-GLI//anti-IgG-HRP	AGA	AMP	0–10 µg/mL	240 ng/mL	[24]
Au/C3SAM/GLI//anti-IgG-HRP	AGA	AMP	0–10 µg/mL	250 ng/mL	[24]
LPFG/(PAH/SM-30)_14_/gliadin	AGA	EWS	n.a.	5 ppm	[25]
OF/(gliadin/PSS)_n_	AGA	EWS	n.a.	1 ppm	[26]
OF/(PAH/PAA)(PAH/PSS)_3_(gliadin/PSS)_2_(gliadin)	AGA	LMR	n.a.	5 ppm	[28]
SPCE/MWCNT/AuNP/DPGx4//anti-IgG-AP	DGPA	CV	n.a.	n.a.	[29]
SPGE/PSS/TG//anti-Ig-HRP	TGA	EIS	n.a.	n.a.	[14]
GEC/tTG//anti-Ig-HRP	TGA	AMP	n.a.	n.a.	[30]
Au/DT2/tTG//anti-IgG-HRP	TGA	AMP	0–10,000 ng/mL	390 ng/mL	[31]
SPCE/MWCNT/AuNP/tTG//anti-Ig-AP	TGA	CV	0–40 U/mL	n.a.	[32]
SPE/MB-TG2//anti-IgA-HRP	TGA	SWV	n.a.	n.a.	[33]
SPCE/tTG//anti-IgG-QD	TGA	DPV	0–40 U/mL	2.2 U/mL	[34]
IDE/cysteamine/tTG//Pr-A-AuNP	TGA	EIS	0.03–30 nM	n.a.	[35]
GCE/OPPy/AuNP/tTG	TGA	EIS	1–100 ng/mL	5.2 ng/mL	[36]
Pt/Ppy-Co(II)SDD/tTG	TGA	EIS	0.2–1.8 µg/mL	201 ng/mL	[37]
SPCE/tTG//anti-IgA-BT/QD-STV	TGA	DPV	3–100 U/mL	2.4 U/mL	[38]
SPCE/tTG//anti-IgA-BT/QD-STV	TGA	DPV	3–40 U/mL	1.0 U/mL	[39]
MWCNT-AuNP/GQD/PAMAM/tTG	TGA	DPV	n.a.	20 fg/mL	[40]
Au/s-tTG//anti-Ig-HRP	TGA	AMP	0.26–6.9 μg/mL	260 ng/mL	[41]
Au/PC/tTG//anti-IgG-SA-Ru	TGA	ECL	1.5–10,000 ng/mL	0.5 ng/mL	[42]
POF/Au/tTG	TGA	SPR	30–3000 nM	n.a.	[43]
Au/MUA/open-tTG//anti-IgG-AuNPs	TGA	QCM	1.3–12 µg/mL	1.3 μg/mL	[44]
Au/ssDNA//ssDNA-HRP	HLA	AMP	1–50 nM	231 pM	[45]
Au/ssDNA//ssDNA-HRP	HLA	AMP	n.a.	n.a.	[46]
AuNP/MB	HLA	FS	0–10 nM	0.5 nM	[47]

^1^ AMP = amperometry, CV = cyclic voltammetry, DPV = differential pulse voltammetry, ECL = electrochemiluminescence, EIS = electrochemical impedance spectroscopy, EWS = evanescent wave absorption spectroscopy, FS = fluorescence spectroscopy, LMR = lossy mode resonance, QCM = quartz crystal microbalance, SPR = surface plasmon resonance, SWV = square wave voltammetry, n.a. = not available.
